# The Degradation Products of Ascorbic Acid Inhibit Amyloid Fibrillation of Insulin and Destabilize Preformed Fibrils

**DOI:** 10.3390/molecules23123122

**Published:** 2018-11-28

**Authors:** Lu-Fei Yang, Cheng-Ming Zeng

**Affiliations:** Key Laboratory of Analytical Chemistry for Life Science of Shaanxi Province, School of Chemistry and Chemical Engineering, Shaanxi Normal University, Xi’an 710119, China; cc6866@hotmail.com

**Keywords:** ascorbic acid, degradation, degradation products, anti-amyloidogenic activity, insulin fibrillation

## Abstract

Ascorbic acid (AsA) is an important antioxidant and enzyme cofactor in many biochemical processes. Most biological activities of AsA are closely related to its redox properties. Recent investigations have demonstrated that AsA is associated with amyloid-related diseases and can inhibit amyloid aggregation of polypeptides. In the present study, we determined the kinetics of AsA degradation and investigated the anti-amyloidogenic activities of AsA and its degradation products by utilizing insulin as a model polypeptide. The results showed that the half-life of AsA varied with the pH of the medium and the incubation temperature. The degradation products of AsA inhibited insulin fibrillation, with an activity positively correlated to the degree of AsA degradation. The degradation species, compared with intact AsA, also showed a stronger disruptive effect on mature amyloid fibrils and significantly decreased fibrillar cytotoxicity. Dehydroascorbic acid and diketogulonic acid, two key intermediates in AsA degradation, had similar anti-amyloidogenic activity toward the degradation species of AsA. The results of this work indicate that degradation of natural antioxidants must be considered when evaluating their anti-amyloidogenic effects. These insights into the action of AsA may also provide a novel route to understand its physiological/pharmacological roles in amyloid-related diseases.

## 1. Introduction

Amyloid fibrillation of protein molecules is associated with a variety of amyloid-related diseases, including Alzheimer’s disease, type 2 diabetes, and several systemic amyloidoses [1,2,3]. These proteins, despite their unrelated amino acid sequences and tertiary structures, can unfold and assemble into fibrils with similar morphologies and β-sheet enriched structures, although the propensities of different sequences to assemble into amyloid fibrils varies widely. Accumulating evidence strongly suggests that amyloid fibrils are cytotoxic, owing to their abilities to disrupt cellular membranes, induce oxidative stress, and trigger a cascade of cellular events leading to apoptosis [4,5]. Recent reports have demonstrated that, in addition to pathogenic isoforms, some amyloid isoforms also have non-pathogenic biological functions [6,7].

Bovine insulin is a polypeptide consisting of a 21-residue A chain and a 30-residue B chain, which are linked by disulfide bonds. Numerous studies have shown that under denaturing conditions, including acidic pH and high temperature, insulin molecules can assemble into amyloid fibrils with a variety of morphologies [8,9,10,11]. Because these fibrils have been well characterized using various methods, insulin is an ideal model system for studying amyloid aggregation and screening amyloid inhibitors. There is no evidence that endogenous insulin forms a pathogenic fibrillar assembly in vivo. However, the fibrillation of non-native insulin causes a variety of problems in the production, storage, and delivery of insulin in pharmaceutical formulations [12,13,14]. An in vivo problem known as injection-localized amyloidosis can occur, triggering an unwanted immune response [12]. Numerous studies have shown that insulin fibrils synthesized in vitro induce the disruption of cell membranes and decrease cell viability through apoptotic and necrotic pathways [15,16].

During the process of amyloid fibrillation, polypeptide chains undergo conformational changes leading to intermolecular interactions coupled with the formation of fibrillar structures [17]. Inhibiting amyloid formation and disrupting formed fibrillar assemblies are the therapeutic strategies proposed for the treatment of amyloid-related diseases. Enzymatic inhibitors, antibodies, peptide fragments, synthetic ligands, and natural antioxidants are among the candidates screened for treating amyloid disorders [18].

Ascorbic acid (AsA), commonly known as vitamin C, is an important biologically active substance acting as an antioxidant and enzyme cofactor in many physiological processes including cell protection, iron absorption, and immune responses [19,20]. As one of the primary water-soluble antioxidants in cells, AsA is a reducing agent and preferentially interacts with oxidants generated during oxidative stress, protecting cell constituents from oxidative damage. Under some circumstances, AsA can act as a pro-oxidant as well [21,22]. Most biological activities of AsA are closely related to its redox property. Accumulating evidence indicates that AsA is associated with amyloid-related diseases [20,23,24], and is capable of inhibiting the amyloid aggregation of polypeptides [25,26,27,28,29,30].

**Scheme 1:**
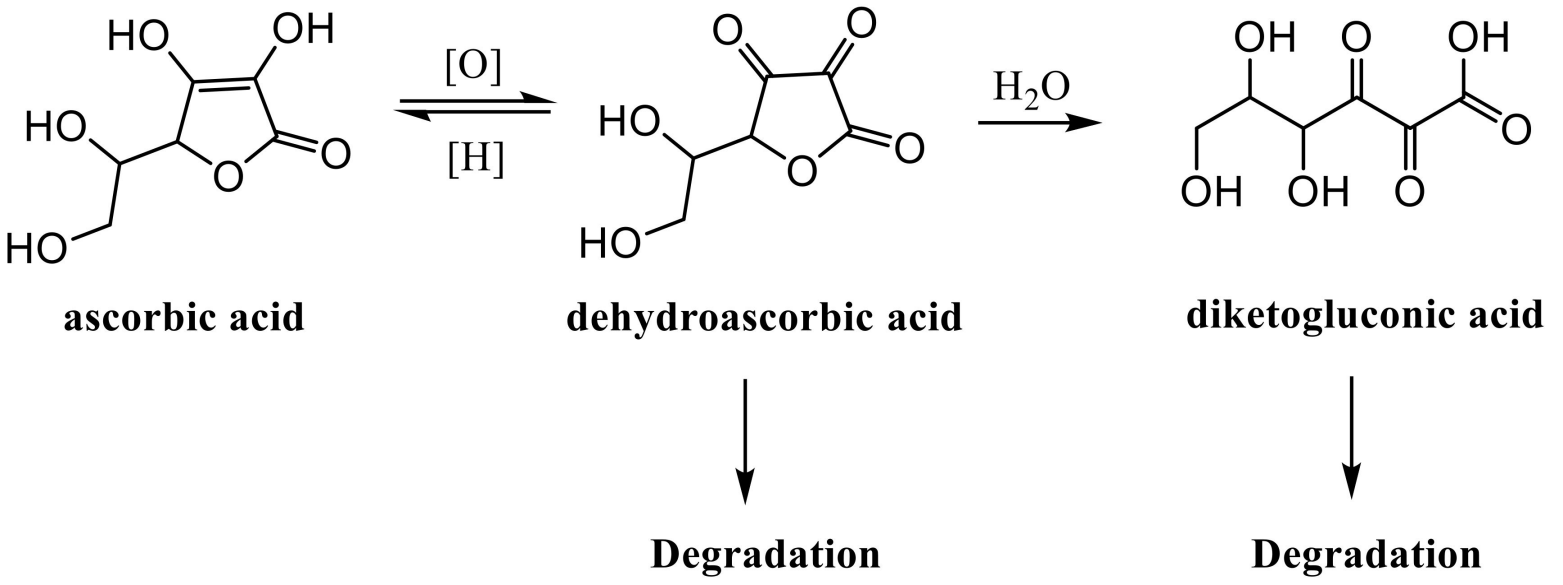
Degradation pathways of ascorbic acid.

As a reducing agent and electron-donor antioxidant, AsA can undergo two consecutive oxidations, thus resulting in the formation of an ascorbate radical and dehydroascorbic acid (DHA). DHA is unstable and is rapidly hydrolyzed to form diketogulonic acid (DKG), transforms reversibly to AsA by reductants, or degrades into oxidative species [19,31]. DKG further converts to a large spectrum of degradation products, depending on the conditions [19,31,32,33] (Scheme 1). Given that AsA is associated with amyloid-related diseases and has anti-amyloidogenic activity, it is critical to explore the roles of AsA and its degradation products in countering amyloid fibrillation. In the present study, we determined the kinetics of AsA degradation in aqueous solutions and investigated the anti-amyloidogenic activities of AsA and its degradation products by utilizing insulin as an in vitro model. The results showed that the stability of AsA varied with the pH of the medium and the incubation temperature. The degradation products of AsA inhibited insulin fibrillation and disrupted preformed mature amyloid fibrils, with an activity positively correlated with the degree of AsA degradation.

## 2. Results

### 2.1. HPLC Analyses of AsA Degradation

**Figure 1:**
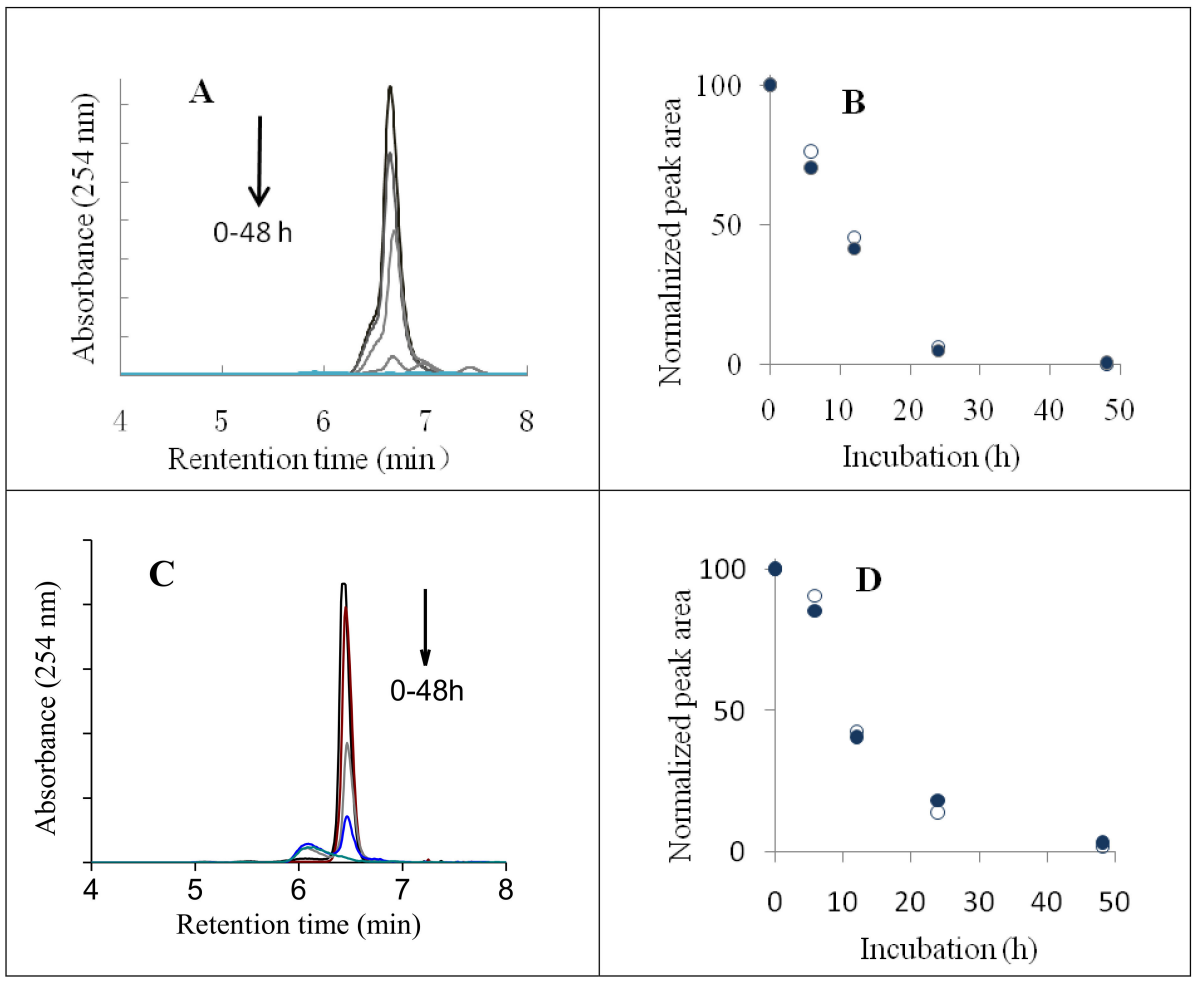
Analysis of AsA degradation. (**A**) HPLC analysis of the kinetics of AsA degradation at 37 °C and pH 7.4, and (**B**) the corresponding time-dependent change in the peak area of AsA. The kinetics of AsA degradation at 60 °C and pH 2.0 and the corresponding time-dependent change in peak area are shown in (**C**) and (**D**), respectively. HPLC analyses were performed in duplicates. All data are shown in (**B**) and (**D**).

AsA was unstable in an aqueous medium. The degradation rate of AsA varied widely with the conditions. To determine the relationship between the degradation and anti-amyloidogenic activity of AsA, we determined the instability of this molecule under the experimental conditions described above. The HPLC data demonstrated that AsA degraded upon incubation at 37 °C in aqueous media at pH 2.0–8.0. AsA was relatively stable in acidic medium, with a maximum half-life of 26.5 h at pH 4.0. The half-life of AsA at pH 7.4 was 10.8 h, as calculated from Figure 1A,B. At pH 8.0, the degradation of AsA accelerated sharply, with a half-life of 3.6 h, thus suggesting that AsA became very unstable in a weak alkali solution. Because amyloid formation was allowed to proceed at 60 °C in 0.01 mM HCl (pH 2.0), AsA degradation under this condition was also determined (Figure 1C,D). The resultant half-life was 11.5 h. We used the degradation products of AsA prepared at pH 7.4 in the following experiments.

### 2.2. Inhibition of Insulin Fibrillation by AsA and Its Degradation Products

Incubation of insulin in an acidic medium under elevated temperature resulted in the formation of amyloid fibrils [10,11,34]. The in vitro synthetic insulin amyloid fibrils shared similar biochemical properties with the fibrils formed by other peptides including Aβ, α-synuclein, and lysozyme. Increasing the temperature, ionic strength, or insulin concentration significantly shortened the lag time of fibril growth. We incubated 0.1 mM insulin in 100 mM NaCl at 60 °C with agitation. The growth of amyloid fibrils was monitored and characterized on the basis of ThT (thioflavin T) fluorescence, ANS (1-anilino-naphthalene 8-sulfonate) fluorescence, and TEM (transmission electron microscopy).

**Figure 2:**
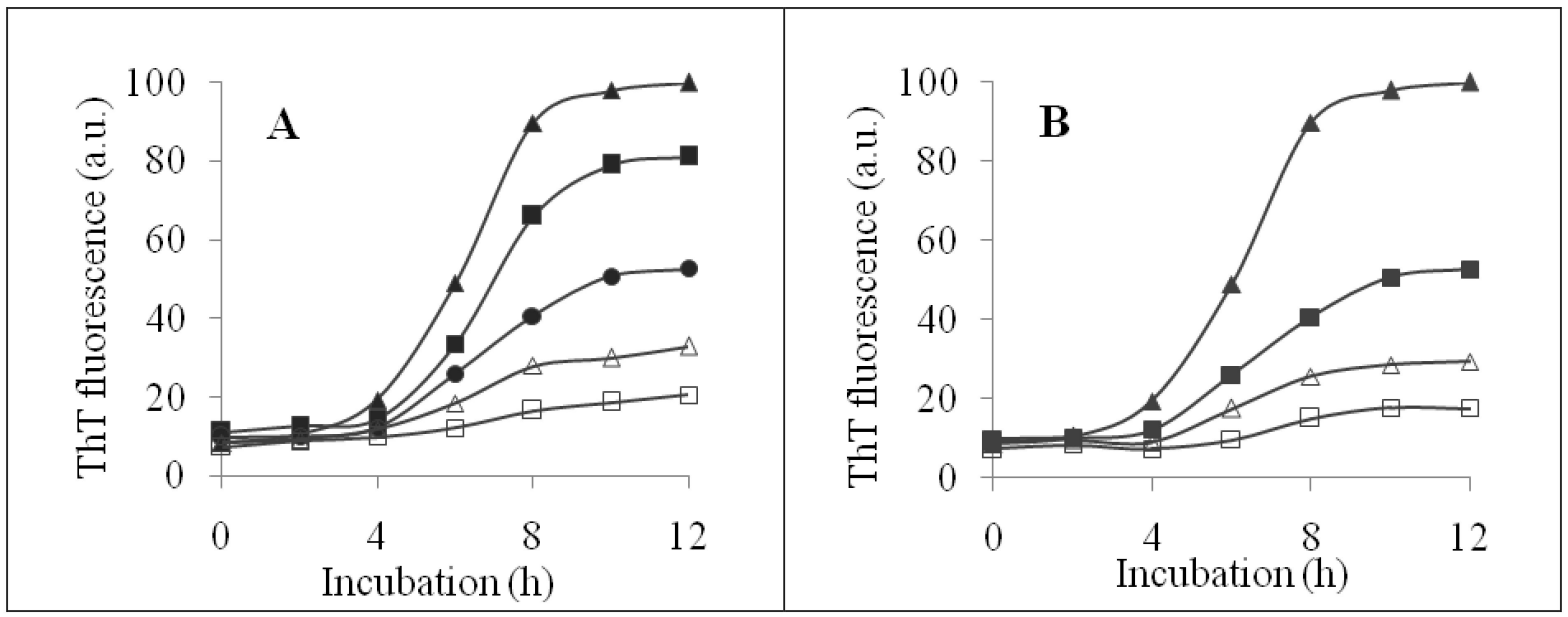
ThT assay to monitor the kinetics of insulin fibrillation in the absence and presence of AsA and its degradation products. (**A**) Dose-dependent inhibitory effects of AsA on insulin fibrillation. Filled triangles, insulin only; filled squares to open squares, insulin incubated with 0.2, 0.4, 0.6, and 0.8 mM AsA, respectively. (**B**) The kinetics of insulin fibrillation in the presence of AsA degradation products. Filled triangles, insulin only; filled squares to open squares, insulin incubated with 0.4 mM AsA degradation products obtained by pre-incubation with AsA for 0, 24, and 48 h, respectively. All data were normalized to the maximum ThT value.

The fluorescence profile of ThT bound to fibrillar insulin was characterized by a lag phase followed by a sigmoid-like elongation phase and a saturation phase, as shown in Figure 2A. AsA inhibited insulin fibrillation in a dose-dependent manner, thus resulting in a significant decrease in the final intensity of ThT fluorescence. The pre-incubated AsA exhibited a noticeably stronger inhibition behavior toward fibril formation than did the freshly prepared AsA. Co-incubation of insulin with 0.4 mM of native AsA resulted in a 46.8% decrease in the final intensity of ThT fluorescence. In the presence of AsA degradation products, pretreatment with AsA for 24 h and 48 h (denoted AsA24h and AsA48h, respectively) resulted in a 71.3% and 80.6% decrease in the final intensity of ThT fluorescence (Figure 2B). Accordingly, the fibril-inhibitory activity of a degradation product was positively correlated with the degree of AsA degradation.

**Figure 3:**
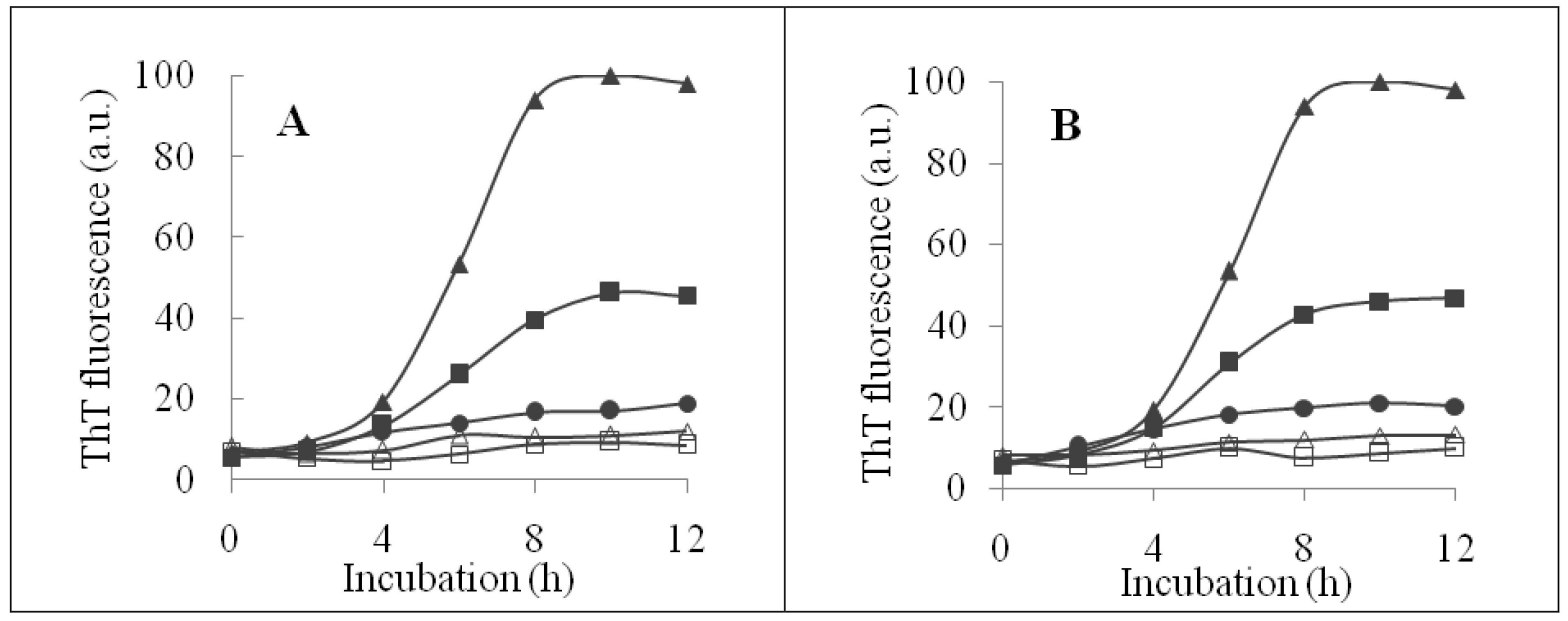
ThT assay to monitor the kinetics of insulin fibrillation in the presence of DHA (**A**) and DKG (**B**). Filled triangles, insulin only; filled squares to open squares, insulin incubated with an inhibitor at a concentration of 0.2, 0.4, 0.6, and 0.8 mM, respectively.

Several substances have been characterized as degradation products of AsA, including DHA and DKG [19,31,32,33]. Next, we determined the inhibitory roles of DHA and DKG on insulin fibrillation. As shown in Figure 3A,B, both DHA and DKG strongly inhibited amyloid formation. Co-incubation of insulin with 0.4 mM of DHA and DKG resulted in an 81.2% and 79.8% decrease in the final intensity of ThT fluorescence, respectively. To compare the inhibitory activities of AsA and its degradation products, the IC_50_ values were calculated according to the ThT data and are listed in Table 1. The results suggested that the degradation products of AsA inhibited insulin fibrillation more strongly than the native form.

Both DHA and DKG were not stable in an aqueous solution. At a neutral pH and 37 °C, the half-lives of DHA and DKG were approximately 25 min and 3 h [35,36]. This implicates that the inhibitory activity of AsA24h and AsA48h on insulin fibrillation come not out of DHA or DKG, but from other degradation species.

DHA and DKG became relatively stable in acidic solutions. Increasing temperature accelerated their degradation. Because amyloid formation was allowed to proceed at 60 °C and pH 2.0, degradation of DHA and DKG under this condition was evaluated. The resultant half-life of DHA was less than 3 h (Figure S1). DKG degraded rapidly and disappeared after 3 h of incubation (Figures S2 and S3). These results suggested that some degradation species of DHA and DKG were active in amyloid inhibition. However, the role of intact DHA and DKG in amyloid inhibition cannot be excluded.

**Figure 4:**
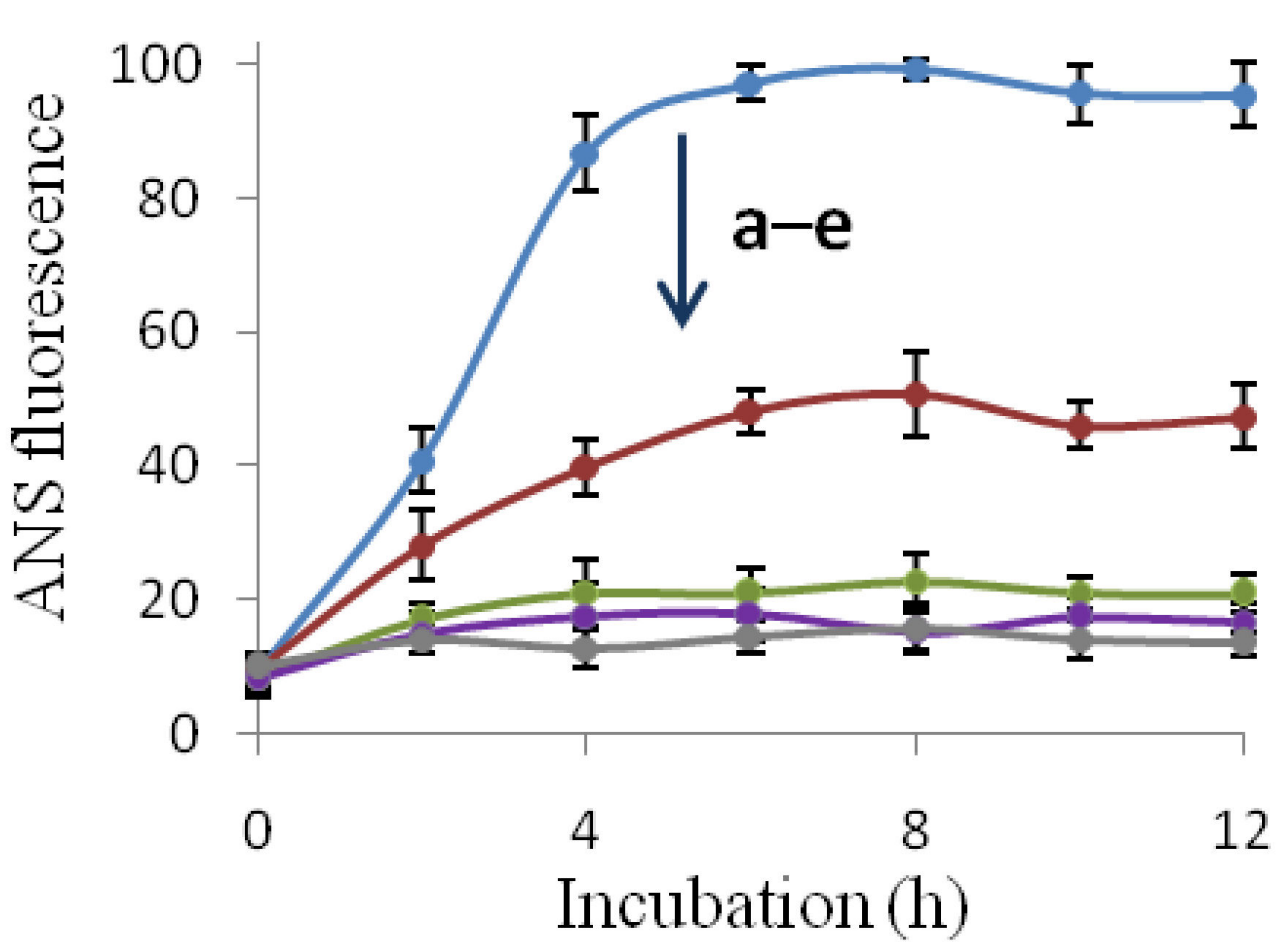
ANS fluorescence of insulin fibril growth in the absence (**a**) and presence of 0.4 mM AsA (**b**), AsA48h (**c**), DHA (**d**), and DKG (**e**). All data were normalized to the maximum ANS value. Data express mean and standard error (n = 3).

ANS is a fluorescent dye used for probing changes in the surface hydrophobicity of protein molecules. Upon ANS binding to a hydrophobic region of protein, the intensity of ANS fluorescence is significantly enhanced, with a blue-shift of the maximum emission wavelength [5]. Incubation with insulin resulted in a significant increase in ANS fluorescence, reflecting an increase in the solvent-exposed hydrophobic interior of the protein during fibril growth. In the presence of native AsA, a significant decrease in ANS fluorescence was recorded, thus suggesting that AsA inhibited exposure of the hydrophobic interior of the protein and subsequently inhibited fibril assembly. The degradation species of AsA were also examined and inhibited the intensity of ANS fluorescence more strongly than native AsA, as shown in Figure 4.

**Figure 5:**
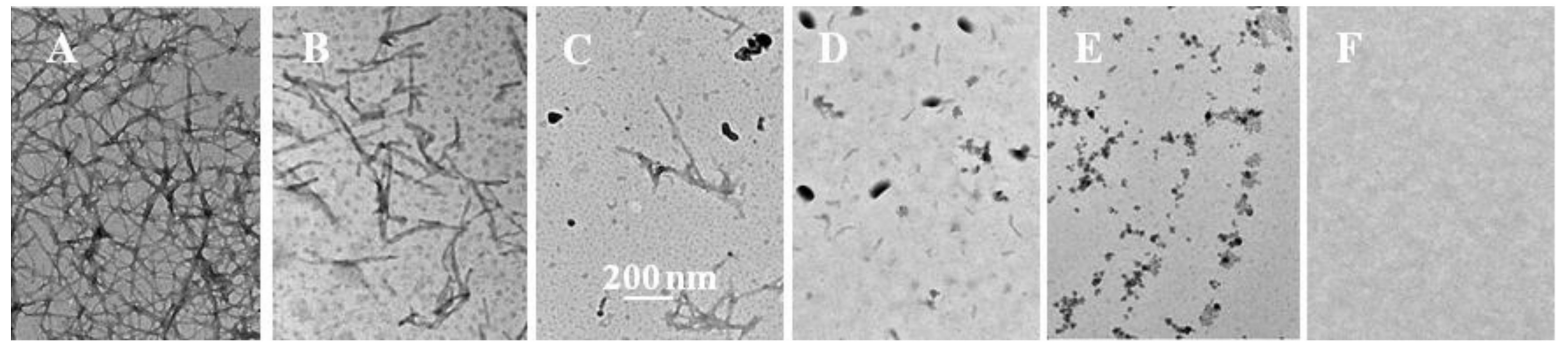
TEM images of insulin assemblies. Samples were prepared via the incubation of insulin (0.1 mM) at 60 °C for 12 h in the absence (**A**) and presence of 0.4 mM AsA (**B**), AsA48h (**C**), DHA (**D**), and DKG (**E**). Insulin (0.1 mM) before incubation is shown in (**F**). Scale bar represents 200 nm.

Figure 5 shows the TEM images of the insulin fibrils prepared in the absence and presence of native AsA and its degradation adducts. In the absence of an inhibitor, the mature insulin fibrils demonstrated a typical amyloid morphology characterized by long, dense, and partially bundled fibrils (Figure 5A). In the presence of 0.4 mM AsA, the length and density of fibrils decreased significantly (Figure 5B). Co-incubation of insulin with the 48-h degradation products of AsA (Figure 5C) and DHA (Figure 5D) resulted in the formation of short fibrils with amorphous aggregates. Amorphous aggregates and probably protofibrils [37] were observed in the sample containing 0.4 mM DKG (Figure 5E). These data indicated that the degradation adducts of AsA, compared with the native form, exhibited a stronger inhibitory role on fibril formation, in agreement with the ThT and ANS results.

### 2.3. The Fibril-Disruptive Roles of AsA and Its Degradation Products

**Figure 6:**
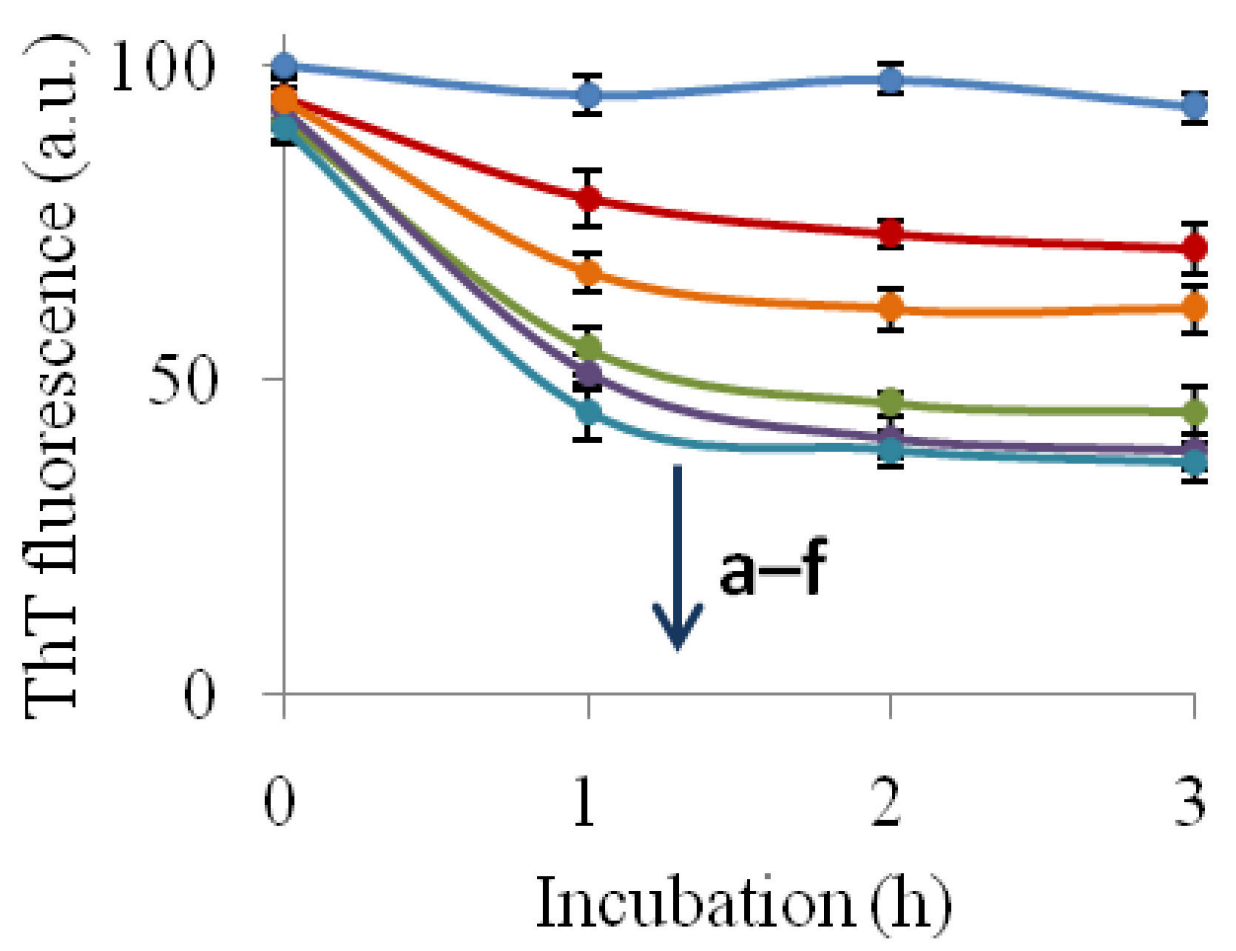
Effects of AsA and its degradation products on the kinetics of fibril destabilization. The reaction mixtures were incubated at 37 °C for the indicated times. Curve (**a**), insulin fibrils only; (**b**–**f**), insulin fibrils incubated with AsA, AsA24h, AsA48h, DKG, and DHA, respectively. All data were normalized to the maximum ThT value, and shown as mean and standard error (n = 3).

Treatment of mature insulin fibrils with AsA or its degradation species resulted in the conversion of amyloid fibrils into amorphous aggregates and a concomitant decrease in ThT fluorescence. As shown in Figure 6, the fluorescence of ThT was nearly unchanged during incubation for 3 h with preformed insulin fibrils without addition of an inhibitor. In the presence of AsA and its degradation species, the ThT fluorescence decreased with time, thus suggesting a disruption of the β-sheet enriched fibrillar structure. When the fibrils were incubated with 0.8 mM AsA, a 29% decrease in ThT fluorescence was observed in 3 h, whereas the results for equimolar concentrations of AsA24h, AsA48h, DHA, and DKG were 39%, 55%, 63%, and 61%, respectively, thus indicating that the degradation species possessed a stronger fibril-disruptive effect than native AsA.

**Figure 7:**
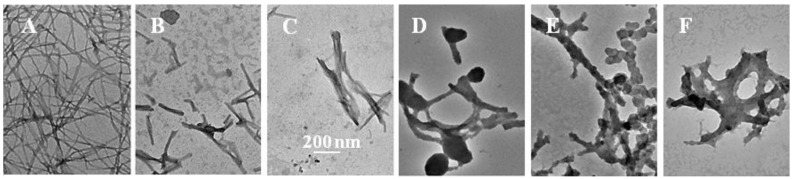
TEM images of insulin aggregates formed by incubating preformed fibrils (0.1 mM) at 37 °C for 3 h in the absence (**A**) and presence of 0.8 mM AsA (**B**), AsA24h (**C**), AsA48h (**D**), DKG (**E**), and DHA (**F**).

Figure 7 shows the TEM images of insulin aggregates induced by treating preformed mature fibrils with native AsA and its degradation products. In the absence of an inhibitor, incubation of mature fibrils for 3 h did not change the fibrillar morphology (Figure 7A). In accordance with the ThT results shown in Figure 6, incubation of insulin fibrils with AsA and its degradation species resulted in a disruption of the fibrillar structure, leading to transformation of the fibrils into short and bundle fibrils (Figure 7B,C), and some short fibrils integrated with amorphous aggregates (Figure 7D–F).

### 2.4. Hemolysis Induced Using Insulin Fibrils Pretreated with an Inhibitor

**Figure 8:**
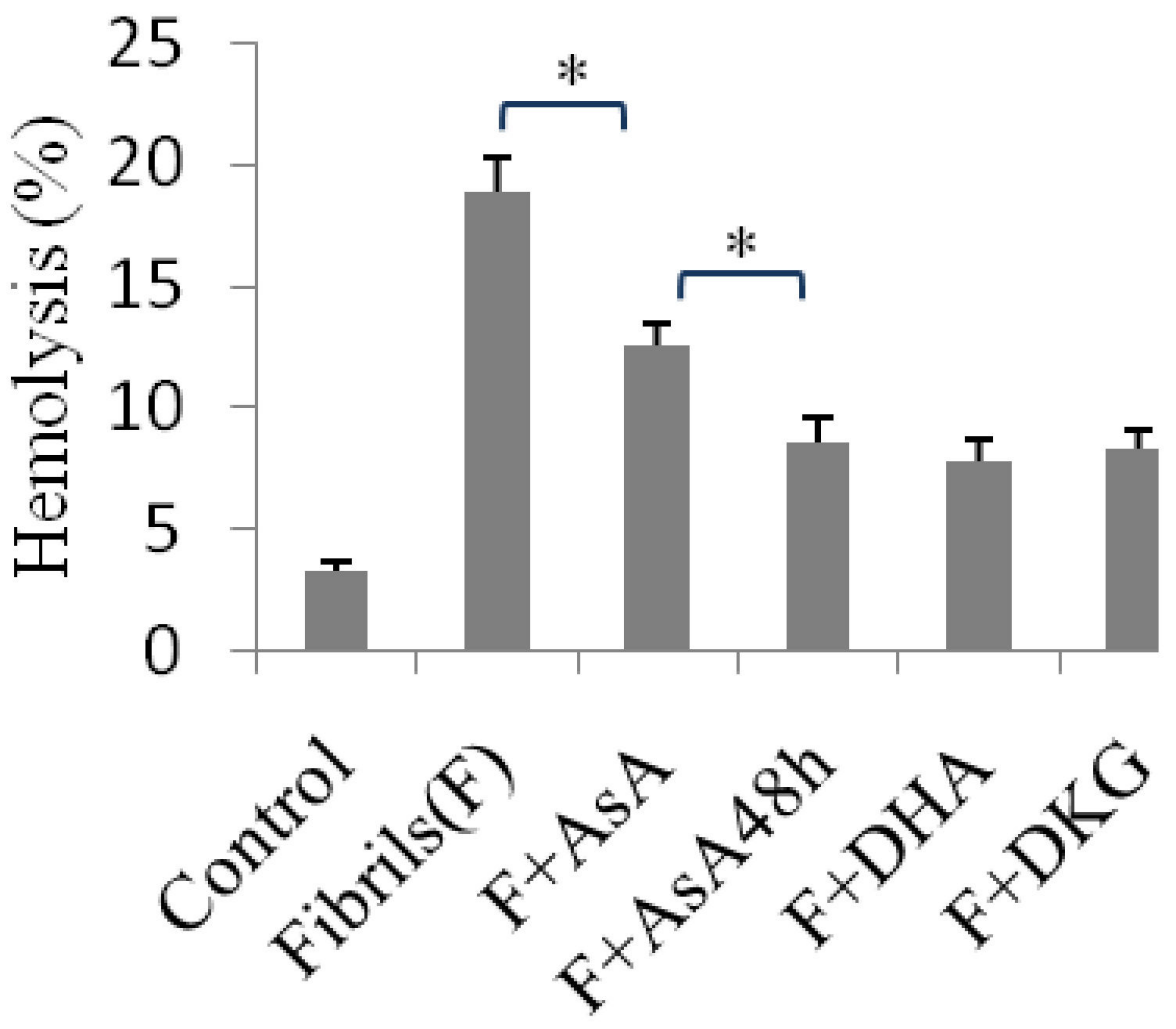
Hemolytic assay. Hemolysis of erythrocytes was induced by incubation of (37 °C) cell suspensions (1%, *v*/*v*) with insulin assemblies for 3 h. Insulin assemblies were prepared by incubation of mature fibrils at 37 °C for 3 h in the absence or presence of AsA and its degradation products. * represents *p* < 0.05 (n = 3).

Previous studies [5,38] have demonstrated that hemolysis can be induced by co-incubating erythrocytes with amyloid fibrils. Under the conditions of the present study, insulin fibrils also exhibited cell-damaging effects. Incubation of erythrocytes in an isotonic environment with mature insulin fibrils resulted in hemolysis. After co-incubation of the cells with insulin assemblies pretreated with AsA or its degradation products, the hemolytic rates decreased significantly (*p* < 0.05), as depicted in Figure 8. In accordance with the data showing fibril disruption, the degradation species of AsA displayed a greater ability than the native form to inhibit fibril-induced hemolysis. For instance, in the presence of AsA48h, the resultant fibrillar assemblies induced 8.6% cell lysis, a value significantly lower (*p* < 0.05) than that (12.5%) for native AsA. This result further supports the conclusion that the degradation species of AsA have stronger anti-amyloidogenic activity than native AsA.

## 3. Materials and Methods

### 3.1. Chemicals

Bovine insulin (5.7 kDa), ascorbic acid, dehydroascorbic acid, dithiothreitol (DTT), thioflavin T ThT, and ANS were purchased from Sigma Aldrich (St. Louis, MO, USA). Other reagents were of analytical grade. Fresh blood was drawn from healthy volunteers by using sodium citrate as an anticoagulant.

DKG was prepared from DHA with alkali treatment according to previously described methods [39]. Briefly, a stock solution of DHA was added with NaOH prior to incubation at ambient temperature. The reaction was then terminated by adjusting the solution to pH 3.5. The freshly prepared DKG solution was stored at 0 °C before assays without further purification.

### 3.2. Stability of AsA

Stock solutions of AsA (80 mM) were prepared daily in double-distilled water prior to dilution with aqueous solution at the desired pH. Incubation was carried out in sealed tubes. The degradation of AsA in aqueous solution was determined with HPLC assays. Chromatographic separation was performed on an Inertsil ODS column (4.6 × 250 mm, 5 μm; GL Sciences, Tokyo, Japan) with a Shimadzu LC-20A system (Kyoto, Japan) at ambient temperature. Samples were filtered through 0.22 μm filters (Merck Millipore, Darmstadt, Germany) prior to injection. The mobile phase consisted of 0.1% formic acid in 40% methanol (HPLC grade) in water. The injection volume was 10 μL, and the flow rate was maintained at 0.5 mL/min in an isocratic elution mode. AsA was detected at 254 nm.

### 3.3. Preparation and Characterization of Insulin Fibrils

Insulin fibrils were prepared according to previously described methods [10,34], with minor modifications. Briefly, insulin was dissolved in HCl solution (10 mM, pH 2.0) containing 100 mM NaCl with or without an inhibitor to a final concentration of 0.1 mM. The mixture was incubated for 12 h at 60 °C in a water bath with agitation (60 rpm). Insulin fibril growth was monitored on the basis of ThT fluorescence, ANS fluorescence, and TEM. For ThT assays, aliquots of insulin fibril solution were diluted with a Tris-HCl buffer (50 mM, pH 8.0), and this was followed by the addition of an aliquot of stock solution of ThT. The final concentration of ThT was 10 μM. ThT fluorescence was measured using a Perkin Elmer LS 55 fluorescence spectrometer (Waltham, MA, USA) with excitation at 440 nm and emission at 485 nm. ThT profiles were representative curves derived from one of three independent experiments. The emission spectra of ANS fluorescence in the presence of insulin fibrils were recorded between 400 and 600 nm using an excitation wavelength of 350 nm [5]. For TEM measurements, an aliquot of insulin fibrils was diluted with water and deposited onto copper mesh grids. Samples were negatively stained with 5% (*w*/*v*) phosphotungstic acid and air-dried at room temperature. Observations were carried out using a JEOL JEM-2100 electron microscope (Tokyo, Japan) with an acceleration voltage of 80 kV.

### 3.4. Fibril-Disruptive Assay

The fibril-disruptive assay was carried out according to methods described previously [40,41] with some modifications. Insulin fibrils (0.4 mM), prepared specifically for the fibril-disruptive assay, were diluted with 50 mM of Tris-HCl (pH 7.4) and supplemented with aliquots of freshly prepared inhibitors prior to incubation at 37 °C. The final concentrations of the fibrils and inhibitor were 0.1 and 0.8 mM, respectively. Aliquots of solution were collected at specific time points for ThT assays.

### 3.5. Hemolytic Assays

Fresh blood was centrifuged at 1000× *g* for 10 min, and erythrocytes were separated from the plasma and buffy coat and washed three times with isotonic phosphate-buffered saline (pH 7.4). For hemolytic assays, cell suspensions (1% hematocrit) were incubated at 37 °C for 3 h in the presence of insulin amyloid aggregates (0.05 mM) obtained by pretreating the fibrils with or without an inhibitor. The molar ratio of insulin to inhibitor was 1:8. After incubation, an aliquot of cell suspension was removed and centrifuged at 1000× *g* for 10 min. The absorbance of the supernatant was determined at 540 nm. The hemolytic rate was calculated in relation to the hemolysis of erythrocytes in 10 mM phosphate buffer (pH 7.4), which was taken as 100%. The control hemolytic rate was obtained in the absence of fibrils and inhibitor. Native insulin, AsA, and all its degradation products showed no obvious hemolytic effects under the experimental conditions described herein.

## 4. Discussion

Extensive studies have described the physiological and pharmacological activities of AsA. Most known physiological and biochemical functions of AsA are due to its action as an antioxidant or prooxidant [19,21]. In the present study, the instability of AsA and the anti-amyloidogenic role of some degradation species of AsA were examined. The results demonstrated that AsA degrades when incubated in aqueous solutions, with a half-life varying with the pH of the medium and the incubation time. The resultant degradation species at neutral pH had a more potent anti-amyloidogenic effect than native AsA.

The degradation of AsA under different circumstances has been investigated extensively. The processes of AsA degradation are very complex and comprise a variety of oxidation/reduction and intermolecular rearrangement reactions [31,32,33,36,42]. Some of the degradation products have been well-characterized. By serving as an electron donor, AsA can readily transform to an ascorbate radical and DHA. The latter can be recycled back to AsA through many mechanisms within cells. DHA is unstable and is easily delactonized to form DKG, which further converts non-enzymatically to a wide range of degradation products, depending on the incubation conditions. Moreover, DHA can be degraded through oxidation to form a number of products. The degradation products of AsA formed via DKG include erythrulose, erythroascorbic acid, and some molecules containing enediol groups or some other functional group that is as easily oxidizable as an enediol group [36,42,43]. These derived substances might function as redox agents similarly to AsA [43].

The results of the present study demonstrated that the degradation products of AsA possessed stronger anti-amyloidogenic activity than the native form, and the 48-h preparations showed anti-amyloidogenic activity similar to that of DHA and DKG. This finding suggests that some degradation species of AsA may play a more important role than the native molecule in amyloid inhibition and fibril disruption. Given DHA and DKG’s instability and complicated degradation processes, a single molecular structure does not appear to be responsible for the anti-amyloidogenic activity. It will be a challenging task to isolate and identify effective amyloid inhibitors from the degradation products of AsA. At present, the possible molecular mechanisms can be tracked in terms of antioxidant activity, and non-covalent and covalent binding of the inhibitors.

Accumulating evidence indicates that antioxidant properties are involved in the anti-amyloidogenic role of some small molecules [25,40,44,45,46,47,48]. For instance, the fibril-disrupting activity of a polyphenol is proportional to its antioxidant capacity [40,48]. In addition, the oxidized form of a polyphenol has been found to have a more potent disruptive effect on amyloid fibrils than the reduced form [38,49,50,51]. Previous studies [52,53] have also found that the inhibition of amyloid fibrillation by polyphenols is associated with quinone formation, and that quinone intermediates are probably the active forms of phenolic compounds that disrupt amyloid structure. Furthermore, oxidative stress has been found to be involved in protein fibrillation and fibrillar cytotoxicity, both in vitro and in vivo [54,55], and a large number of antioxidants can act as anti-amyloidogenic agents through attenuating oxidative stress [54,56,57,58]. This may also be one of the molecular mechanisms underlying the anti-amyloidogenic actions of AsA and its degradation species. Indeed, AsA itself and many AsA degradation species can scavenge reactive oxygen species during amyloidogenic processes. However, how an inhibitor exerts its anti-amyloidogenic role through its antioxidant activity is not clear and requires further exploration.

Numerous investigations have suggested that covalent binding of a small molecule to a peptide chain can alter the interacting forces of peptide monomers and/or oligomers, thus leading to interruption of amyloid fibrillation and disaggregation of preformed fibrils [38,50,51,52,53,59]. Most of the active substances originating from AsA degradation, including ascorbate radicals, DHA, DKG, erythrulose, and others, are highly reactive and can covalently modify polypeptides or induce cross-linking of protein molecules. To verify whether insulin was modified via AsA and its degradation species during incubation, we performed mass spectrometry. The results indicated that no adducts of insulin were generated after incubation of the polypeptide with the inhibitors (Figure S4).

In general, the anti-amyloidogenic effects of small molecules are attributed to their direct interaction with folded or misfolded polypeptides in a non-covalent manner. Hydrogen bonding, hydrophobic interactions, and aromatic stacking have been suggested to be driving forces underlying the inhibitors’ anti-amyloidogenic roles [57,58,59,60,61,62]. Previous reports have attributed the anti-amyloidogenic effect of AsA mainly to the hydrogen bonding between peptide chains and the inhibitor [26,27,28,29,30]. Hydrophobic and electrostatic interactions between the inhibitor and amino acid residues have also been suggested to be involved in amyloid inhibition and fibril disruption [27,30]. A common structural feature of AsA degradation species is the presence of multi-hydroxyl groups. Upon interaction with amyloidogenic peptides/proteins, these molecules exert their roles through hydrogen bonding, including preventing the conformational transition of the peptide to a cross-β-sheet fibrillar structure, thereby interfering with the aggregation pathway and disassembling formed amyloid fibrils. In addition, other functional groups, such as keto/diketo, enediol, and carboxyl moieties, may also play important roles in the anti-amyloidogenic activity of AsA degradation species.

In summary, AsA was susceptible to degradation under the experimental conditions of this study. The half-life of AsA varied with the pH of the medium and the temperature of incubation. By utilizing insulin as an in vitro model, we found that the degradation products of AsA had a more potent inhibitory role toward amyloid formation than the native molecule, and its activity was positively correlated with the degree of AsA degradation. The degradation species also had a stronger disruptive effect on preformed amyloid fibrils than intact AsA, thus resulting in a significant decrease in fibrillar cytotoxicity. DHA and DKG, two key intermediates generated during AsA degradation, possessed similar anti-amyloidogenic activity to the 48-h degradation species of AsA. Because DHA and DKG are also susceptible to degradation, the exact effective chemical structure of the AsA degradation species and the molecular mechanism underlying the anti-amyloidogenic action still remain to be further investigated. Increasing numbers of natural antioxidants are being reported to inhibit amyloid formation and disrupt preformed amyloid fibrils. A common property of these substances is their susceptibility to oxidation or degradation. The results of this work indicate that the degradation of natural antioxidants cannot be neglected in assessing their anti-amyloidogenic effects. These insights into the action of AsA may provide a novel route to understanding its roles in amyloid-related diseases.

## Figures and Tables

**Table 1:** IC_50_ values (mM) of substances exhibiting inhibition of insulin fibrillation.

	AsA	AsA24h	AsA48h	DHA	DKG
IC_50_	0.43 ± 0.07	0.29 ± 0.04 *	0.20 ± 0.03 ^#^	0.17 ± 0.03 ^##^	0.18 ± 0.03 ^##^

Data express mean and standard error (n = 3). * represents p < 0.05 vs. the value of AsA; ^#^ p < 0.05 and ^##^ p < 0.01 vs. the value of AsA24h.

## Supplementary Materials

The supplementary materials are available online.
