# Supplementary material for: The Degradation Products of Ascorbic Acid Inhibit Amyloid Fibrillation of Insulin and Destabilize Preformed Fibrils
Source: Molecules. 2018 Nov 28;23(12):3122. doi: 10.3390/molecules23123122 (PMC6320921; doi:10.3390/molecules23123122)

Supplementary Material

The Degradation Products of Ascorbic Acid Inhibit Amyloid Fibrillation of Insulin and Destabilize Preformed Fibrils

1. *Determination of DHA and DKG (Methods)*

DHA in solution has little absorbance above 220 nm. In this work, DHA was determined indirectly according to the spectrophotometric method described previously (Gómez Ruiz, et al. 2016) with minor modification. The method is based on the reduction of DHA into AsA. Briefly, the reduction was carried out by mixing 0.5 mL of sample with 0.25 mL of 60 mM DTT aqueous solution and 0.25 mL 100 mM phosphate buffer (pH 7.4). The mixture was homogenized and kept dark for 30 min. DTT in excess was eliminated by three successive extractions with ethyl acetate. AsA concentration was determined at 254 nm. The absorbance value was converted into DHA concentration using a calibration curve of DHA obtained the same day.

DKG has its absorption maximum at 300 nm (Figure S1), and therefore can be analyzed by HPLC. For other HPLC conditions, see Materials and Methods (2.2).

2. *MALDI–TOF MS analysis of insulin (Methods)*

Insulin fibrils/aggregates were prepared by incubation of insulin (0.1 mM) at 60 ºC for 12 h in the absence or presence of 0.8 mM inhibitor, according to the method described in Materials and Methods (2.3). The resultant fibrils/aggregates were separated using a Hitachi CS150GXL micro-

ultracentrifuge at 200,000 *g* for 40 min. The supernatant was obtained and subject to dialysis and ultrafiltration prior to MS analysis. MALDI–TOF MS analysis was performed on a Bruker microflex system (Bruker Daltonics Inc., USA) in a positive-ion reflector mode with a scan range of 4000–8000 m/z.

Reference

1. Gómez Ruiz, B.; Roux, S.; Courtois, F.; Bonazzi, C. Spectrophotometric method for fast quantification of ascorbic acid and dehydroascorbic acid in simple matrix for kinetics measurements, *Food Chemistry* **2016**, *211*, 583–589.

Figure S1. DHA degradation at 60 ºC and pH 2. Fresh prepared DHA were diluted to 0.8 mM prior to incubation at 60 ºC and pH 2 for 12 h. Samples were collected at specific time points for spectrometric analysis (n=3).

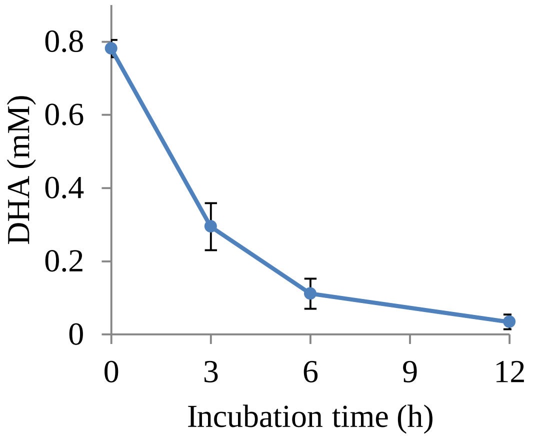

Figure S2. UV spectra of DKG. Fresh prepared DKG were diluted to 0.2 mM in 10 mM HCl prior to spectral scanning at 200–400 nm by using a U3900/3900H spectrophotometer (Hitachi, Tokyo, Japan).

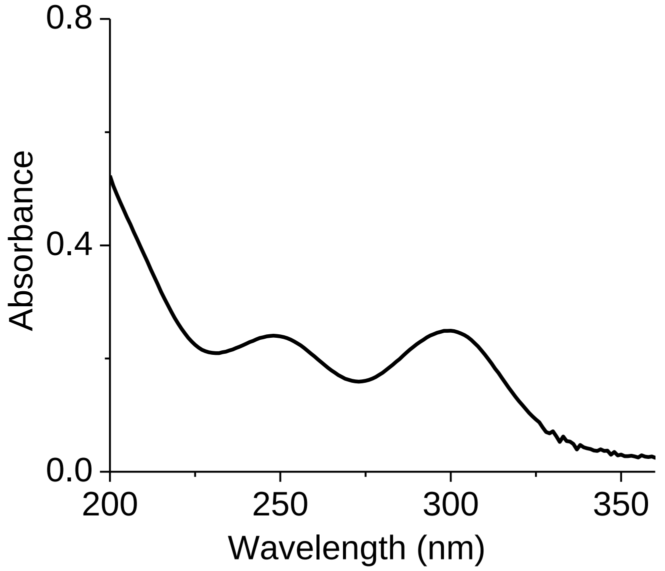

Figure S3. DKG degradation at 60 ºC and pH 2. Fresh prepared DKG were diluted to 0.8 mM prior to incubation at 60 ºC and pH 2 for 12 h. Samples were collected at specific time points for HPLC analysis.

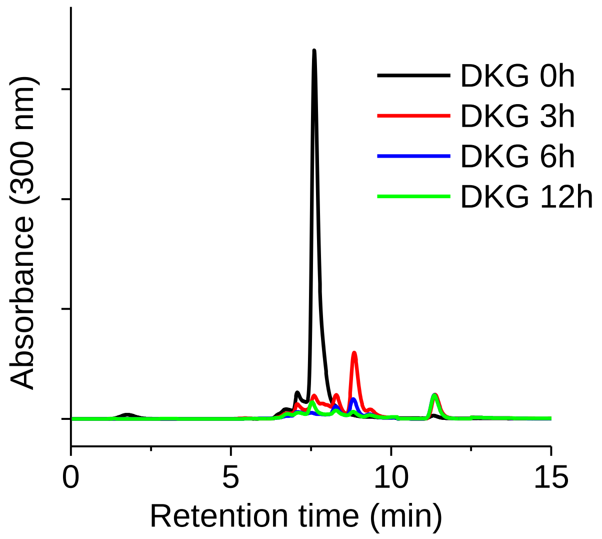

Figure S4. MALDI–TOF MS analysis of insulin monomer. Insulin fibrils/aggregates were prepared by incubation of insulin (0.1 mM) at 60 ºC for 12 h in the absence (A) and presence of 0.8 mM AsA48h (B). Other inhibitors showed similar effect to that of AsA48h.

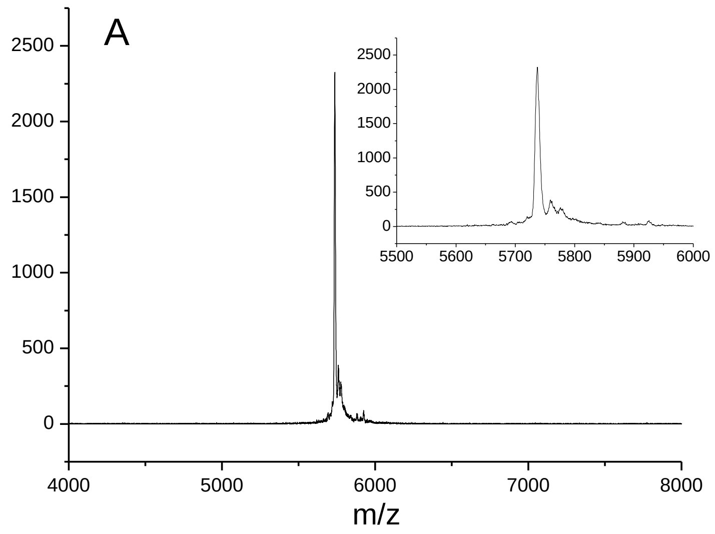

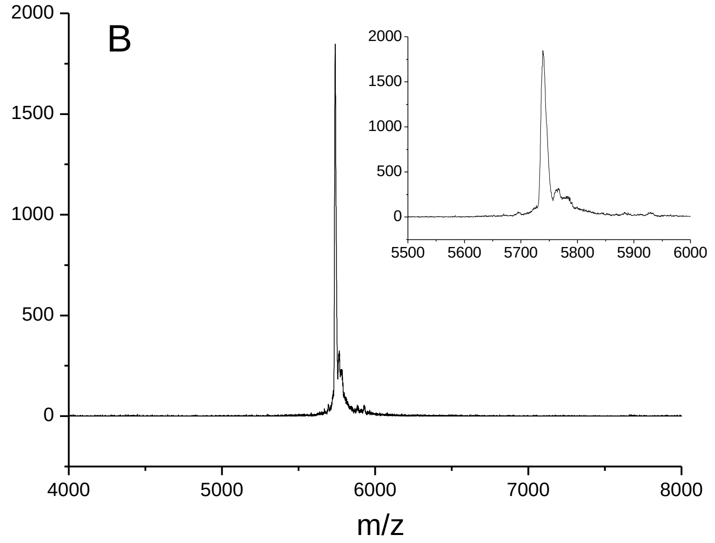

Supplement: Supplementary file 1 [file molecules-23-03122-s001.pdf]
